# The Effect of Parental Control on Cyber-Victimization in Adolescence: The Mediating Role of Impulsivity and High-Risk Behaviors

**DOI:** 10.3389/fpsyg.2019.01159

**Published:** 2019-05-22

**Authors:** David Álvarez-García, José Carlos Núñez, Paloma González-Castro, Celestino Rodríguez, Rebeca Cerezo

**Affiliations:** Department of Psychology, University of Oviedo, Oviedo, Spain

**Keywords:** parental control, cyber-victimization, impulsivity, high-risk internet behaviors, adolescence

## Abstract

The aim of this work is to analyze the relationship between parental control and cyber-victimization in adolescence, considering the possible mediating effect of impulsivity, and high-risk internet behavior. To that end we analyzed the responses of 3360 adolescents aged between 11 and 18 (*M* = 14.02; *SD* = 1.40), from Asturias (Spain), to four previously validated questionnaires in order to measure the level of parental control over the use of the internet (restriction and supervision), along with high-risk internet behaviors, impulsivity, and cyber-victimization in the adolescents. The results show that parental control tends to have a protective effect on the likelihood of the children being victims of cyber-aggression, with impulsivity, and high-risk internet behaviors as mediating variables. More specifically, parental restriction and supervision are positively related to each other; both forms of parental control are negatively related with the adolescent’s engaging in high-risk internet behaviors; supervision is negatively related with impulsivity; impulsivity is positively related with high-risk internet behaviors; and both impulsivity and high-risk internet behaviors are positively related to being a victim of cyber-aggression. The practical implications of these results are discussed.

## Introduction

The mobile phone and the internet can be very positive tools for adolescents’ development, allowing them to keep in touch with family and friends, and offering many learning opportunities. However, they can also be very dangerous if they are used to cause harm. The term *cyber-aggression* is commonly used to refer to acts which intentionally harm or offend via electronic communication devices. *Cyber-victimization* refers to being a victim of those aggressions (Álvarez-García et al., 2018b).

At this moment in time, there is great social concern about this problem, because of its prevalence, and effects. Different studies offer data which varies greatly about prevalence, depending on the characteristics of the samples being analyzed and the methodology used. It is estimated that between 4.9 and 65% of adolescents have been victims of aggression via electronic media (Brochado et al., 2017) and that between 2 and 7% have suffered severe cyber-aggression (Garaigordobil, 2011). Cyber-victimization can have serious consequences for the victim, especially in severe cases. It has mainly been associated with an increase in internalizing problems, suchas anxiety (Rose and Tynes, 2015), low self-esteem (García et al., 2015), social anxiety (Juvonen and Gross, 2008), depressive symptomatology (Bonanno and Hymel, 2013), and suicidal ideation (Van Geel et al., 2014).

For this reason, it is important to have strategies which can effectively combat the problem (Díaz-Lopez et al., 2019). In order to do that, the principal associated protective and risk factors need to be identified. Parents are often advised to exercise a certain *control* over their children’s use of mobile phones and the internet in order to prevent them from becoming victims of cyber-aggression. Families often set limits or *restrictions* on internet and mobile use (time, content, activities, and contacts), whether by setting rules or by using specific software; or they *supervise* their children’s activity either openly or surreptitiously, during or after the activity.

However, research attempting to analyze the relationship between both forms of parental control (restriction and supervision) and cyber-victimization in adolescence is scarce and has produced inconsistent results. Some studies have found a negative relationship between the two forms of parental control (restriction and supervision) and cyber-victimization, which is greater for supervision but is small in both cases (Khurana et al., 2015). Other studies have not found statistically significant relationships between parental control and becoming a victim of cyber-aggression: neither for restriction, such as installing filters or software that blocks websites (Navarro et al., 2013), nor for supervision, such as checking the web pages that children visit on the internet (Navarro et al., 2013) or direct parental monitoring of internet use (Mishna et al., 2012). A third group of studies suggests that the relationship between the two forms of parental control and cyber-victimization is positive (Álvarez-García et al., 2015; Sasson and Mesch, 2017; Wright, 2017; Wright and Wachs, 2018). This might be explained by a tendency of some parents to exercise more control if they know that their children are suffering cyber-victimization or think that there is a risk that they will suffer from it, or because the family rules are not combined with parental support (Martins et al., 2016). A lack of parental warmth (support, dialogue, open communication, trust, affective relationships, and parental interest in children’s activities) increases the probability of suffering from cyber-aggression (Elsaesser et al., 2017; Gómez-Ortiz et al., 2018). All of these results suggest a complex relationship between parental control and cyber-victimization. Some research suggests that the impact of parental control on cyber-victimization is indirect and in order to understand it, the intermediate variables that modulate its effect need to be understood.

One intermediate variable that seems important, according to previous research, is adolescents engaging in high-risk behavior on the internet. Some studies indicate that parental control could be a protective factor for high-risk behavior, such as intensive internet use (Chen and Chng, 2016; Gómez et al., 2017; Villanueva-Blasco and Serrano-Bernal, 2019), having Internet access in the bedroom (Khurana et al., 2015), or disclosing personal information (Liu et al., 2013). Other studies show that engaging in these high-risk behaviors on the internet increases the likelihood of becoming a victim of cyber-aggression (Helweg-Larsen et al., 2012; Sasson and Mesch, 2014) such that parental control would be expected to be a protective factor for cyber-victimization through its protective effect on high-risk internet behavior. Nevertheless, some studies have produced apparently contradictory results: adolescents who received higher levels of restrictive parental mediation (Shin and Ismail, 2014) and supervision (Sasson and Mesch, 2014) were more inclined to engage in risky online activities. If parental control is excessive or is imposed in a climate of little affection or communication it may be counterproductive in terms of engaging in risky behavior (Sasson and Mesch, 2014; Shin and Ismail, 2014).

The relationship between parental control and high-risk behavior may be mediated by impulsivity. Some studies indicate that teaching rules and parental supervision are protective factors for impulsivity in children (Li et al., 2014; Chen and Chng, 2016; Kurtz and Zavala, 2017), especially if they occur in a context of parental warmth (Ruiz-Hernández et al., 2019). Impulsivity is, in turn, positively related to engaging in high-risk internet behavior. Adolescents with poor self-control spent much more time on the Internet (Li et al., 2014) and made more self-disclosures (making personal or even private information public) on the Internet (Yu, 2014). Nonetheless, this relationship is also complex and is modulated by other variables. Some studies highlight that personal risky information may not only be published impulsively and spontaneously, but rather in a planned way, in order to improve a person’s social image on the web, and people may be well aware of the potential risk of publication (White et al., 2018).

**FIGURE 1:**
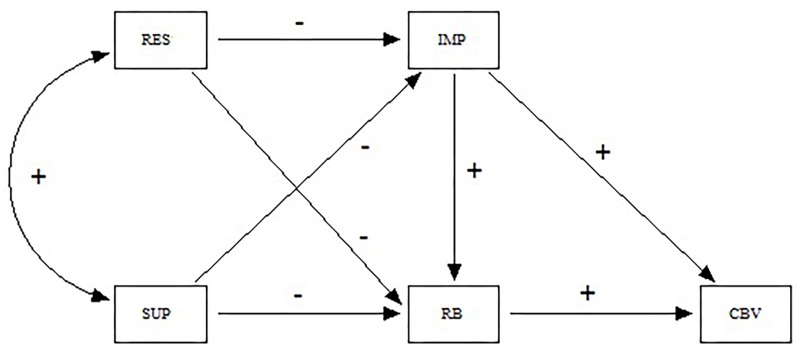
Starting theoretical model (RES, restriction; SUP, supervision; IMP, impulsivity; RB, risk behaviors; CBV, cyber-victimization; +, positive relation; -, negative relation).

In sum, previous research suggests a complex relationship between parental control and cyber-victimization, although the precise mechanisms by which that happens are still not clear. This leads to the objective of our study: to analyze the relationship between parental control and cyber-victimization in adolescence, considering the possible mediating effect of impulsivity, and risky internet behavior. If we consider previous research, we expect the theoretical model in Figure 1 to have a good fit to the empirical data.

## Materials and Methods

### Participants

Students from twenty schools were selected by a combination of stratified and cluster random sampling from all Compulsory Secondary Education schools supported by public funds in Asturias (Spain). The population of schools was divided according to type (public or semi-private), and a number of schools proportional to the population were randomly selected from each group. The questionnaires were given to all students in the 1st to 4th years of Compulsory Secondary Education in each selected school, totaling 3360 students, aged between 11 and 18 years old (*M* = 14.02; *SD* = 1.40). Of them, 48.3% were girls.

### Measuring Instruments

#### Parental Control for Adolescent Internet Use Questionnaire (Álvarez-García et al., 2019)

This is made up of 7 items. For each item, the respondent indicates the extent to which they think the corresponding statement about possible control of their Internet use by their parents is true. The questionnaire measures two types of control: restriction [3 items; α = 0.70; e.g., “En casa me han puesto algunas normas sobre lo que puedo o no puedo hacer en Internet” (“At home my parents have set some rules about what I can or can not do on the Internet”)] and supervision [4 items; α = 0.80; e.g., “Cuando accedo a Internet en mi tiempo libre, mis padres me vigilan y echan un vistazo a la pantalla” (“When I access the Internet in my spare time, my parents watch me and take a look at the screen”)]. The responses are in a Likert-type format with four alternatives (from 1 = completely false to 4 = completely true). In both types of parental control the total score for each respondent corresponds to the sum of the scores on each item (restriction: theoretical minimum 3, maximum 12; supervision: minimum 4, maximum 16). Higher scores indicate greater control by families.

#### High-Risk Internet Behaviors Questionnaire (Álvarez-García et al., 2018a)

This is a self-report made up of 8 items, each of which describes a high-risk behavior on the internet [e.g., “Suelo publicar información personal en mis redes sociales: qué voy a hacer, dónde y con quién; fotos o vídeos personales o familiares;..” (“I usually publish personal information on my social networks: what I am going to do, where, and who with; personal or family photos or videos…”)]. The respondent indicates the extent to which they think it is true that they engage in each of the behaviors through a Likert-type scale with four alternatives (from 1 = completely false to 4 = completely true). The total score for each respondent in this factor corresponds to the sum of the scores on each item (minimum 8 and maximum 32). High scores indicate that the respondent engages in a lot of high-risk internet behaviors. The internal consistency of the scale in this study sample is adequate (α = 0.73).

#### Impulsivity Scale (Álvarez-García et al., 2016a)

This was created using part of the impulsivity criteria proposed by the DSM-5 (American Psychiatric Association [APA], 2013) for the diagnosis of Attention Deficit and Hyperactivity Disorder. It consists of five items [e.g., “A menudo contesto antes de que se haya completado la pregunta” (“I often answer before the question has finished”)] with a Likert-type response scale with four options (from 1 = completely false to 4 = completely true). The total score for each respondent in this factor corresponds to the sum of the scores on each item (minimum 5 and maximum 20). High scores indicate high levels of impulsivity. The internal consistency of the scores obtained with the scale in this study sample is adequate (α = 0.75).

#### Cyber Victimization Questionnaire for Adolescents (CYVIC; Álvarez-García et al., 2017)

This measures the frequency with which the respondents report having been victims of aggression via mobile phone or the Internet during the last 3 months [e.g., “Se han burlado de mí con comentarios ofensivos o insultantes en las redes sociales” (“Someone has made fun of me with offensive or insulting comments on social networks”)]. It consists of 19 Likert-type response format items (from 1 = never to 4 = always). In this study, the total score in cyber-victimization for each respondent was obtained by adding the scores from the 19 items (minimum 19 and maximum 76). High scores indicate high levels of cyber-victimization. The internal consistency of the scale in this study sample is adequate (α = 0.79).

### Procedure

Permission to administer the questionnaires was requested from the administration in each school selected. Each school obtained family consent for the participation of the students in the study because they were underage. The questionnaires were completed by the students at the school during normal school hours. At the time of the application of the questionnaires, participants were informed of the voluntary and anonymous nature of the test as well as the confidential treatment of the data obtained.

### Data Analysis

Preliminary analysis was performed to examine the mean, standard deviation, skewness and kurtosis for each variable included in the starting theoretical model. The relationship between these variables was analyzed by using the Pearson correlation coefficient or the Spearman correlation coefficient depending on whether the variable scores were normally distributed or not. Following that, path analysis was used to examine how well the starting theoretical model fit the empirical observed data. Given the non-normality of the data (Mardia = 14.44), Robust Maximum Likelihood was used as the method of estimation. To determine the degree of fit of the tested models, the Chi-square (χ2)/degrees of freedom (df) ratio, the comparative fit index (CFI), the Bentler-Bonett non-normed fit index (NNFI), the standardized root mean square residual (SRMR), and the root mean square error of approximation (RMSEA) were utilized. Usually, the fit is considered good when CFI ≥ 0.95, NNFI ≥ 0.95, SRMR ≤ 0.08, and RMSEA ≤ 0.06 (Hu and Bentler, 1999), and χ2/df < 3 (Ruiz et al., 2010). The analyses were carried out using the statistical programs SPSS 24 (IBM Corp, 2016) and EQS 6.2 (Bentler, 2014).

## Results

The study participants tended to give low scores in the five variables included in the starting theoretical model (restriction, supervision, impulsivity, high-risk internet behaviors, and cyber-victimization). This tendency was especially marked in the case of cyber-victimization, which was the only variable whose distribution was significantly far from normality (Table 1). All of the correlations between the model variables were statistically significant (Table 1).

**Table 1:** Descriptive statistics and correlation coefficients between the variables in the starting theoretical model.

	1	2	3	4	5
1. Restriction					
2. Supervision^1^	0.616^∗∗∗^				
3. Impulsivity^1^	-0.110^∗∗∗^	-0.165^∗∗∗^			
4. High-risk behavior^1^	-0.231^∗∗∗^	-0.272^∗∗∗^	0.350^∗∗∗^		
5. Cyber-victimization^2^	-0.069^∗∗∗^	-0.090^∗∗∗^	0.292^∗∗∗^	0.388^∗∗∗^	

Mean	6.00	7.67	10.27	13.67	21.57
Standard deviation	2.75	3.62	3.49	4.42	3.29
Response range	3–12	4–16	5–20	8–32	19–57
Skewness (*SE* = 0.04)	0.60	0.76	0.45	0.87	2.77
Kurtosis (*SE* = 0.09)	-0.75	-0.59	-0.38	0.43	13.30


^*1*^Pearson correlation coefficients. ^*2*^Spearman correlation coefficients. ^∗∗∗^*p* < 0.001.

**FIGURE 2:**
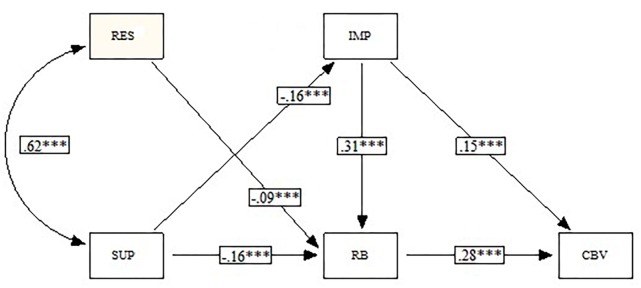
*Post hoc* path model (RES, restriction; SUP, supervision; IMP, impulsivity; RB, risk behaviors; CBV, cyber-victimization). ^∗∗∗^*p* < 0.001.

The starting theoretical model (Figure 1) showed a good fit to the empirical data [SBχ^2^ = 8.34; df = 2; SBχ^2^/df = 4.17; CFI = 0.997; NNFI = 0.986; SRMR = 0.016; RMSEA = 0.033 (90% CI 0.012–0.057)]. However, the effect of restriction on impulsivity was not statistically significant (Standard Error = 0.029; Critical Ratio = -0.667; *p* > 0.05). Consequently, the path analysis was repeated after removing this effect (Figure 2).

The path analysis performed (Figure 2) demonstrated a positive correlation between the two forms of parental control (restriction and supervision). Both forms of parental control had a direct and negative effect on engaging in high-risk internet behaviors. High-risk internet behaviors had a positive, direct effect on cyber-victimization. Therefore, both forms of parental control had an indirect and negative effect on cyber-victimization through their effect on engaging in high-risk internet behaviors. High-risk internet behaviors constitute a mediating variable between both forms of parental control and cyber-victimization.

Supervision had a direct and negative effect on impulsivity. Impulsivity had a positive, direct effect on cyber-victimization, but mainly indirect through its effect on high-risk internet behaviors. Therefore, supervision had an indirect and negative effect on cyber-victimization through its effect on impulsivity. Impulsivity constitutes a mediating variable between supervision and high-risk internet behavior, as well as between supervision and cyber-victimization.

The effects were statistically significant but small, except for the relationship between restriction and supervision, and the effect of impulsivity on high-risk internet behavior, which were moderate (Figure 2). The fit of the model to the obtained empirical data in the study was good [SBχ^2^ = 8.97; df = 3; SBχ^2^/df = 2.99; CFI = 0.997; NNFI = 0.991; SRMR = 0.016; RMSEA = 0.026 (90% CI 0.007–0.046)].

**FIGURE 3:**
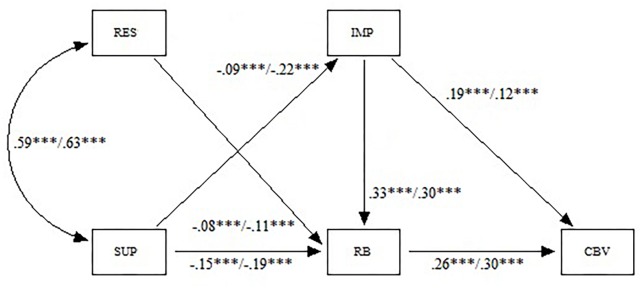
Result of the path analysis performed with boys (left) and girls (right) (RES, restriction; SUP, supervision; IMP, impulsivity; RB, risk behaviors; CBV, cyber-victimization). ^∗∗∗^*p* ≤ 0.001.

No appreciable differences in the predictive capacity of the variables were observed between boys and girls (Figure 3). Both in boys as in girls, the fit of the model was good. Boys: SBχ^2^ = 3.12; df = 3; SBχ^2^/df = 1.04; CFI = 1.00; NNFI = 1.00; SRMR = 0.013; RMSEA = 0.005 (90% CI 0.000–0.044). Girls: SBχ^2^ = 5.14; df = 3; SBχ^2^/df = 1.71; CFI = 0.998; NNFI = 0.994; SRMR = 0.017; RMSEA = 0.022 (90% CI 0.000–0.054).

## Discussion

The aim of this study was to analyze the relationship between parental control and cyber-victimization in adolescence, considering the possible mediating effect of impulsivity and high-risk internet behaviors. The results agree with the starting theoretical model (Figure 1), excepting for the direct effect of restriction on impulsivity. More specifically, parental restrictions and supervision are positively related with each other; both forms of parental control are negatively related with the adolescent’s engaging in high-risk internet behaviors; supervision is negatively related with impulsivity; and both impulsivity and high-risk internet behaviors are positively related to falling victim to cyber-aggression.

In this study, restriction and supervision exhibit a moderate level of co-occurence. This suggests that although setting rules and monitoring are often complementary, they are different entities and do not always happen concurrently. In this study, the participants reported that their parents place few restrictions and do little supervision of the use they make of the internet, which is in line with previous research (Rial et al., 2014; Arnaiz et al., 2016).

According to the results we obtained, the two forms of parental control (restriction and supervision) exhibit a protective effect on engaging in high-risk internet behaviors, although very small in the case of restriction and small in the case of supervision. One possible reason for this weak influence of parental control is that peers take on greater importance during adolescence (Cutrín et al., 2017). It is difficult for parents or guardians to exercise rigorous control over adolescents’ use of the internet and mobile phones. Adolescents spend a lot of time away from their parents and they might go online out of sight or using different devices (Álvarez-García et al., 2015). In addition, the peer group often encourages or approves of risky behavior more than families (Sasson and Mesch, 2014; Shin and Ismail, 2014). Although parents may set sufficient restrictions, occasionally peer pressure may encourage an adolescent to break the rules and engage in risky behavior.

The results we obtained suggest that supervision is also more effective than restriction in order to prevent impulsivity in the adolescents (and consequently, high-risk behavior, and cyber-victimization). Impulsivity is a risk factor for cyber-victimization, both directly and indirectly via its effect on high-risk internet behaviors. This suggests that impulsive adolescents may be victims of cyber-aggression due to their tendency to high-risk internet behaviors, but also due to off-line acts where their impulsivity might have a prominent role. The relationship between risky behavior and cyber-victimization in the path analysis was statistically significant but small. One possible explanation is that it is not strictly necessary to engage in high-risk internet behavior (or use the internet at all) to become a victim of certain types of cyber-aggression (Álvarez-García et al., 2015).

This research contributes to the study of the complex relationship between parental control and cyber-victimization in adolescence. From a theoretical perspective it helps to clarify the mechanisms behind this relationship. From a practical perspective it offers some clues towards more effective prevention of this problem. Parental control has a protective effect on cyber-victimization, albeit limited and indirect. In order for parental control to be an effective protective factor, it must happen in an environment of parental affection, and communication (Elsaesser et al., 2017; Gómez-Ortiz et al., 2018). There are various reasons for this. Firstly, restrictions that are simply imposed without debate, or at the very least explanation, and excessive supervision can be perceived by adolescents as interference in their ongoing search for autonomy from their parents and can therefore end up being counterproductive (Sasson and Mesch, 2014; Shin and Ismail, 2014). They can cause conflicts and make communication and togetherness harder for parents and children (Sasson and Mesch, 2014). A good climate of family affection and communication facilitates self-disclosure by adolescents. This is a subtle form of control, consisting of spontaneous revelation by the child to their parents about what they do in their free time, and is generally the consequence of a climate of affection and communication between parents and children (Álvarez-García et al., 2016b). If there is a good family atmosphere, adolescents will feel comfortable sharing what they do and what happens to them with their parents, which is a protective factor against high-risk behavior in the children (Urry et al., 2011). Secondly, restrictions themselves only aim to avoid risky situations. They do not require a discussion between the parents and children about the right way to act online and the possible risks, nor do they teach strategies to face issues when they occur. This discussion and anticipation of negative consequences may prevent impulsive behavior and subsequently reduce the risk of cyber-victimization (Wright, 2017). Thirdly, excessive parental control has a negative impact on other variables that are also related to risky behaviors and cyber-victimization, such as self-esteem and shyness/social anxiety (Álvarez-García et al., 2015). Finally, supervision and good communication mean that it is easier for parents to be aware of the applications their children are using, which helps give better recommendations for their proper use and better supervision. The applications that adolescents use change constantly. One of the things which contributes to parental control having a limited protective effect is the difficulty parents face in having the same level of knowledge and understanding of new technologies (Shin and Ismail, 2014).

Despite the contributions of this study, it is not without its limitations. In the first place, it was carried out with a large, random sample of adolescents but constrained in terms of age and specific geographical context. This means that any generalization of the results of this study to other ages and contexts should be made with care. In the future, it would be interesting to replicate this study with other ages and in other contexts. Secondly, the only measuring instruments were questionnaires directed at students. It would be useful to complement that in the future with data gathered using other techniques (e.g., interviews or discussion groups) and informants (particularly parents). Thirdly, this was a transversal study. It would be interesting to test whether the hypothesized causal relationships would be confirmed in longitudinal studies. Finally, the model we tested did not consider the role of other potentially important variables, which might be mediators, or modulators of the relationships examined in this study. For example, previous studies have highlighted the importance of peer influence and school climate as predictors of cyber-victimization (Zych et al., 2019).

## Ethics Statement

This study was carried out in accordance with the recommendations of the Deontology Commission of the General Counsel of Psychology of Spain, with written informed parental consent for all subjects. All subjects family gave written informed consent in accordance with the Declaration of Helsinki. The protocol was approved by the research and ethics committee at the University of Oviedo.

## Author Contributions

DA-G and JN designed the study, analyzed the data, and drafted the manuscript. PG-C, CR, and RC critically reviewed the draft and made significant contributions to the final version.

## Conflict of Interest Statement

The authors declare that the research was conducted in the absence of any commercial or financial relationships that could be construed as a potential conflict of interest.
